# Lestaurtinib potentiates TRAIL‐induced apoptosis in glioma via CHOP‐dependent DR5 induction

**DOI:** 10.1111/jcmm.18077

**Published:** 2023-12-10

## Abstract

Yingxiao Cao, Shiqi Kong, Yuling Xin, Yan Meng, Shuling Shang, Yanhui Qi. Lestaurtinib potentiates TRAIL‐induced apoptosis in glioma via CHOP‐dependent DR5 induction. *J Cell Mol Med*. 2020; 24: 7829–7840. https://doi.org/10.1111/jcmm.15415.

The above article, published online on 22 May 2020 in Wiley Online Library (wileyonlinelibrary.com), has been retracted by agreement between the authors, the journal Editor‐in‐Chief, Stefan Constantinescu, The Foundation for Cellular and Molecular Medicine, and John Wiley and Sons Ltd. The retraction has been agreed upon following an investigation into concerns raised by a third party, which revealed inappropriate duplications of images between this and other articles that were either previously published or published later in the same year in a different scientific context. Thus, the editors consider the conclusions of this manuscript substantially compromised.

Yingxiao Cao, Shiqi Kong, Yuling Xin, Yan Meng, Shuling Shang, Yanhui Qi. Lestaurtinib potentiates TRAIL‐induced apoptosis in glioma via CHOP‐dependent DR5 induction. *J Cell Mol Med*. 2020; 24: 7829–7840. https://doi.org/10.1111/jcmm.15415.

The above article, published online on 22 May 2020 in Wiley Online Library (wileyonlinelibrary.com), has been retracted by agreement between the authors, the journal Editor‐in‐Chief, Stefan Constantinescu, The Foundation for Cellular and Molecular Medicine, and John Wiley and Sons Ltd. The retraction has been agreed upon following an investigation into concerns raised by a third party, which revealed inappropriate duplications of images between this and other articles that were either previously published or published later in the same year in a different scientific context. Thus, the editors consider the conclusions of this manuscript substantially compromised.

